# Recent Trends in Potential Therapeutic Applications of the Dietary Flavonoid Didymin

**DOI:** 10.3390/molecules23102547

**Published:** 2018-10-06

**Authors:** Qing Yao, Meng-Ting Lin, Yin-Di Zhu, He-Lin Xu, Ying-Zheng Zhao

**Affiliations:** School of Pharmaceutical Sciences, Wenzhou Medical University, Wenzhou 325035, China; yqpharm@163.com (Q.Y.); lmtpharm@163.com (M.-T.L.); zhuyindi314@sina.com (Y.-D.Z.); xhlpharm1214@126.com (H.-L.X.)

**Keywords:** didymin, dietary flavonoid glycoside, therapeutic effects, cancer, neurodegenerative disorders

## Abstract

Didymin (isosakuranetin 7-*O*-rutinoside) is an orally bioactive dietary flavonoid glycoside first found in citrus fruits. Traditionally, this flavonoid has long been used in Asian countries as a dietary antioxidant. Recent studies have provided newer insights into this pleiotropic compound, which could regulate multiple biological activities of many important signaling molecules in health and disease. Emerging data also presented the potential therapeutic application of dietary flavonoid glycoside didymin against cancer, neurological diseases, liver diseases, cardiovascular diseases, and other diseases. In this review, we briefly introduce the source and extraction methods of didymin, and summarize its potential therapeutic application in the treatment of various diseases, with an emphasis on molecular targets and mechanism that contributes to the observed therapeutic effects. The dietary flavonoid didymin can be used to affect health and disease with multiple therapeutic targets, and it is anticipated that this review will stimulate the future development of this potential dietary medicine.

## 1. Introduction

Flavonoids are a type of plant secondary metabolites widely found in various edible plants and largely characterized by a common benzo-γ-pyrone structure. Many citrus species accumulate large amounts of flavonoids during the development of their various organs [1,2,3]. Simmons et al. have reviewed the production, consumption and health benefits of citrus [4], which is detailed and comprehensive. Flavonoids have attracted the interest of researchers because of their demonstrated biological therapeutic properties in various disease prevention and treatment [5,6,7,8]. Flavonoids involved in the aging process, triggering the anti-oxidative activities and circumvent oxidative stress, tissue damage, and inflammatory process [9].

Moreover, the available evidence indicates that flavonoids such as luteolin and quercetin are important anticancer substances with multiple effects [10,11]. Some flavonoids, such as ginkgo flavonoid extract, baicalin, exhibited desired neuroprotective effects and had been exploited as a competitive alternative medicine for the treatment of ischemic nerve damage [12]. However, the mechanism underlying the neuroprotection is not clear, but plainly relevant to the increased cerebral blood flow, reduced ischemia-related cell apoptosis, and resulted in increased neuronal viability. Dietary flavonoids could also affect the ability of human platelets to aggregate and capillary fragility, and participate in antioxidant and immune regulation mechanisms [13,14]. 

Structurally, flavonoids contain C_6_–C_3_–C_6_ backbone skeletons derived from phenylpropanoids. Moreover, based on the heterocyclic C-ring, flavonoids fall into six major subclasses [15], namely flavan-3-ols, flavones, flavanones, anthocyanidins, flavonols, and isoflavones. Core unit modification, e.g., hydroxylation, methylation, prenylation, alkylation, and glycosylation also greatly amplify the number of these flavonoids. Flavonoid glycosides are natural molecules in which flavonoids are bond to sugar via a glycosidic bond. They are a dominant existential form of flavonoids and a common component of many plants.

**Figure 1:**
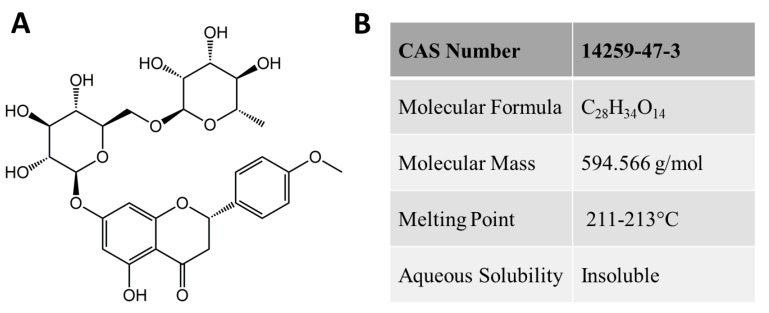
Structure and basic properties of didymin. (**A**) The chemical structure of didymin. (**B**) Major physical and chemical properties.

Didymin (systematic name (*S*)-7-((6-*O*-(6-Deoxy-alpha-l-mannopyranosyl)-β-d-gluco-pyranosyl)oxy)-2,3-dihydro-5-hydroxy-2-(4-methoxyphenyl)-4*H*-benzopyran-4-one), is a typical dietary glycoside also known as neoponcirin and isosakuranetin-7-*O*-rutinoside (Figure 1). Didymin is commonly found in citrus fruits and campanula, including mandarin, bergamot, orange, *Origanum*, and *Vulgare Duanxueliu*. Due to its high content in citrus and easy extraction, didymin has been recognized as an inexpensive, safe and effective oral drug that does not cause toxicity to normal tissues [16]. The therapeutic potential of didymin with anti-oxidant in promoting health is drawing more attention in recent five years. With the intensive pharmacological study results, didymin seems to have more important prospects other than a pure natural antioxidant. For example, Hung et al. demonstrated the anticancer effect of didymin and provided evidence that didymin can cause cell death in non-small cell lung cancer cells [17].

In this review, we will briefly introduce the source and extraction methods of natural didymin from plants. We highlight the potential therapeutic applications of didymin in cancer, neurological diseases, liver diseases, cardiovascular diseases, and other diseases. More importantly, detailed therapeutic action mechanisms of didymin, including newly identified signaling pathways targeting, are discussed and enlightens future development of this potential dietary medicine. 

## 2. Source, Extraction and Detection Method

Didymin is a dietary flavanone glycoside distributed in plants such as citrus and campanula. It is a white needle-like compound that has a higher solubility in methanol than water, and it should be stored in a cool, dark place. Currently, didymin is reported as one of the most common flavonoids, including citrus fruits [18] and Chinese herbs [19]. For example, “Valencia” oranges contained 3.42 mg/g, 14.5 mg/g, and 1.62 mg/g for narirutin, hesperetin and didymin, respectively [20]. Chaudhary et al. reported that didymin is ranking fourth on the levels of health-promoting flavonoids in Rio Red grapefruit with a content of 2.49 ± 0.10 mg/g [21]. Didymin also be rich in *Clinopodium chinense*, also known as “Duanxueliu”, which is a traditional Chinese herb for the treatment of hematuria, skin trauma, influenza and allergic dermatitis [22].

Along with the potential use of the main components of flavonoids in citrus are more recognized, more extraction and detection methods have been applied in the studies [23]. The natural source and extraction separation method of didymin are summarized in Table 1. High performance liquid chromatography (HPLC) is a commonly used method for separating and extracting compounds. It has been shown that HPLC can separate and purify citrus flavonoids from juice [24,25]. Researches using HPLC method for the separation of flavanone glycosides and polymethoxylated flavones in citrus fruits [26]. This technique enable to quantitate six 12 different type flavonoids, including didymin. Sun et al. reported that using an HPLC with photodiode array detection could simultaneously determine several flavonoids, including namely, naringin, hesperidin, didymin, tangeretin and nobiletin, from different parts of citrus reticulata “Chachi” fruit [27]. Moreover, mass spectrometry-HPLC (MS-HPLC) systems with high selectivity and sensitivity are often used as detection methods for flavonoids [28,29]. In the past few years, ultra-HPLC (UHPLC) has been described for the determination of flavanones in citrus juices [30,31,32]. 

Furthermore, some applications on the analysis of citrus flavonoids by comprehensive multidimensional HPLC methods have also been reported [33]. Calabrò developed a reversed-phase HPLC (RP-HPLC) separation using photodiode array detection for the simultaneous determination of flavonoids extracted from the citrus dipping sauce [23]. In this study, HPLC method, employing a C18 reversed-phase column and a linear gradient elution system with methanol/water (*v*/*v*) as the mobile phase, the detection wavelength of 283 nm, is used to separate and extract didymin [23]. Rocco et al. used a nano-liquid chromatography ultraviolet–visible (nano-LC/UV-Vis) apparatus to analyze and quantify the major flavanones in citrus juices. Nano-scale LC system coupled with a mass spectrometer, the low flow rate, and corresponding low solvent consumption is the most significant advantage of this methodology, the technique is environmentally friendly, it can be identified quickly and accurately analyze flavonoids [34].

Recently, Hernández extracted phenolic antioxidants from red corncob using ultrasound-assisted extraction (UAE) [35]. Moreover, Wojtanowski has reported the development of HPLC combined with electrospray ionization (ESI) octapolar quadrature time-of-flight (TOF) MS to separate phenolic acids, flavonoids, sesquiterpenes, etc [36]. The Cudalbeanu group performed ultrasonic separation and HPLC-MS/MS identification of polyphenols and flavonoids in a Danube Delta biosphere extract for the first time [37]. At present, a variety of HPLC analytical methods for flavonoids can also be used for didymin. 

## 3. Therapeutic Bioactivities: Protective Effects and Health Benefits

**Figure 2:**
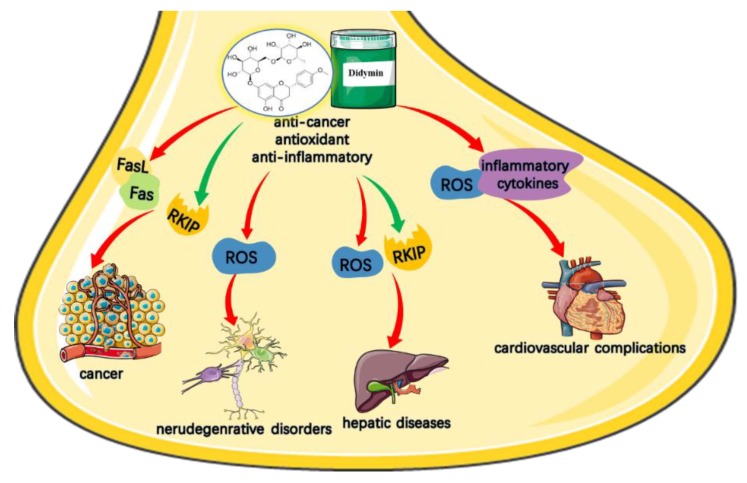
Therapeutic application of didymin in promoting the health. The green arrow indicates the promoted effect of didymin. The red arrow indicates the inhibitory effect of didymin.

Many plants, fruits, and their products are known to have health benefits for human and have been used for the prevention and treatment of many diseases since ancient times. Citrus plants, e.g., oranges, grapes, mandarins, limes, and lemons, are rich in various flavonoids, which possessing various biological activities as is well known. In recent years, studies have confirmed that didymin as a flavonoid showed extensive inhibition of oxidative stress and affected cell proliferation and invasion pathways to anticancer. Also, Morelli et al. first discovered that didymin could protect nerve cells from oxidative damage [42]. Moreover, flavonoids may have therapeutically potential in the treatment of inflammation-related diseases as cytokine modulators [43]. Similarly, didymin is also reported to be involved in modulating the immune system as an anti-cancer agent in cancer treatment. Many molecular mechanisms have been proposed and elucidated to confirm the potential activities of didymin. In this regard, more studies have focused on didymin and its multiple therapeutic targets, but in vivo data is scarce. Only limited studies with detailed pharmacokinetic studies and in vivo activities of didymin have been performed. Most research is limited to in vitro studies on cellular physiology and lack sufficient information on the in vivo data and practical formulation strategies. In this part, we will discuss recent advances in understanding the therapeutic effects of didymin. We have summarized the potential application of didymin for various diseases (Figure 2), including cancer, neurodegenerative diseases, cardiovascular complications, and its underlying molecular mechanisms with various signaling modulations (Table 2), which could enlighten future development of this potential dietary medicine.

### 3.1. Didymin and Anti-Tumor Property

Recent reports have shown that eating vegetables and fruits in the diet can reduce the risk of cancer [17]. Natural dietary flavonoids, unlike toxic chemotherapeutic agents, are better tolerated and less toxic to humans. Studies showed that co-delivery of such products with chemotherapeutics provides superior anti-tumor efficacy [44]. For example, apigenin, a kind of flavonoid, could sensitize tumor cells to classic chemotherapeutic (e.g., paclitaxel)-induced apoptosis by superoxide dismutase (SOD) activity downregulation, reactive oxygen species (ROS) accumulation and caspase-2 cleavage [45]. Chakrabarti et al. also investigated combination cancer therapy with two plant-derived flavonoids, luteolin and silibinin [46]. Their results showed that the natural flavonoid combination provided an effective treatment inhibiting cell migration and inducing apoptosis in different glioblastoma cells and stem cells.

Didymin has been proved to possess therapeutic effects on different types of tumors, such as lung cancer, breast cancer, and brain tumor. Unlike traditional chemotherapeutics, the molecular mechanism of this anti-tumor effect is still unclear. In most cases, didymin served as an inhibitor of proliferation of different classes of cancers, including apoptosis and death, by several different signaling pathways. Herein, we will look insight into the therapeutic potential of didymin in cancer prevention and treatment, and discuss the involved molecular mechanisms.

Lung cancer is one of the leading causes of cancer death, and the majority of all lung cancer cases are non-small cell lung cancers (NSCLCs) [47,48]. NSCLC is generally resistant to radiotherapy and chemotherapy, compromising anti-tumor treatment efficacy. Moreover, detection usually occurs too late for patients to undergo surgical intervention for NSCLC due to its aggressive progression, resulting in an overall five year survival rate of less than 15% [49]. The lack of safe and effective treatments has compelled the researchers to look for the new options to reduce the incidence of lung cancer and improve the overall therapeutic options.

**Figure 3:**
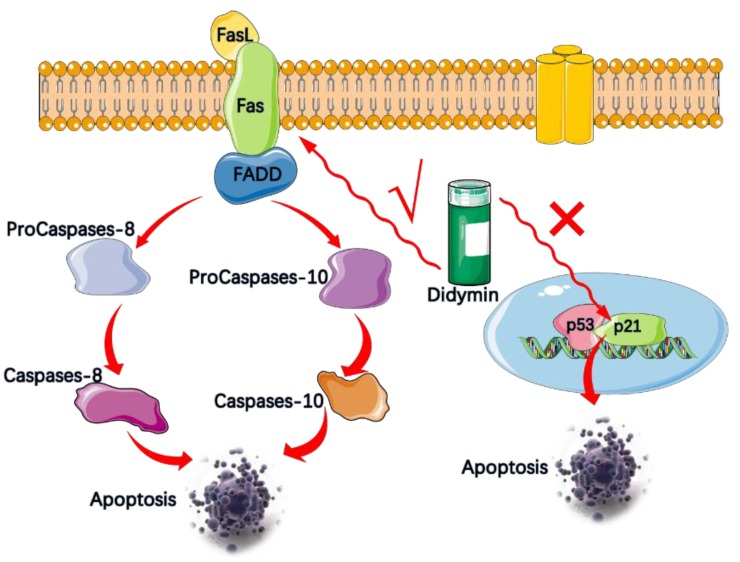
Didymin is involved in lung cancer cell signaling pathways. The main pathway of apoptosis of A549 and H460 cells induced by didymin is the Fas/Fas ligand apoptotic system. Fas is a cell surface receptor when its ligand (FasL) recognizes and activates Fas; it leads to oligomerization of the intracellular death domain and recruitment of the intracellular adaptor Fas-associated death domain (FADD). After binding, FADD can activate procaspase-8 and procaspase-10 in the death-inducing signaling complex, causing A549 and H460 cells apoptosis or death without the mediation of p53 and p21/WAF1.

Epidemiological studies have indicated that high levels of flavonoids, fruit and vegetable intake might reduce the prevalence of cancer in humans. Hung et al. first investigated the potential anti-cancer properties of the dietary flavonoid glycoside didymin in human NSCLC cancer cells in vitro and in vivo [17]. Didymin showed a significant antiproliferative effect in lung cancer cells in a dose-dependent manner. The half maximal inhibitory concentration (IC_50_) values of didymin were 12.57 μM and 11.06 μM in A549 and H460 cancer cells [17]. To clarify the underlying antitumor properties of didymin, mechanism of action studies, including apoptosis, cellular cycle distribution, and cellular signaling were carefully carried out (Figure 3). Many flavonoids induce apoptosis in cancer cells by mediating p53 and p21/WAF1 [50,51]. However, Hung’s group confirmed that the primary pathway of apoptosis in lung cancer cells induced by didymin is the Fas/Fas ligand apoptosis system. The Fas/FasL system has been recognized as a key signal transduction pathway of cellular apoptosis [52]. To be more specific, Fas is a cell surface and its ligand (FasL) could recognize and activate Fas, which leads to oligomerization of the intracellular death domain and recruitment of the intracellular adaptor Fas-associated death domain (FADD). After binding, FADD can activate procaspase-8 and procaspase-10 in the death-inducing signaling complex, causing A549 and H460 cells apoptosis or death without the mediation of p53 and p21/WAF1 (Figure 3). More importantly, in vivo study showed that 6 mg/kg/day of didymin significantly suppress tumor growth without detectable side effects in tumor-bearing mice [17]. Harvested A549 tumor xenografts at the end of in vivo study revealed that increased didymin-mediated apoptosis correlated with the results of the in vivo anti-tumor study, therefore, didymin is involved in the Fas/FasL apoptotic system in the anti-proliferative effects of cancer cells, resulting in increased apoptosis and apparent anti-tumor property. However, whether this pro-apoptotic effect contributes to the potential chemotherapy effect in fighting NSCLC still requires future clinical research in human patients. 

Neuroblastoma is a malignant brain tumor derives from primitive neural crest cells [3]. Different ages, sites of tumors, and different degrees of tissue differentiation can lead to considerable differences in its biological characteristics and clinical manifestations. Some neuroblastomas can naturally subside or be converted into benign tumors, but others are hard to treat and have a poor prognosis [53]. One primary genetic causes of high incidence and refractory treatment of neuroblastoma include the continuous expansion of the oncogene N-Myc and the deletion of the tumor suppressor p53 [54]. 

**Figure 4:**
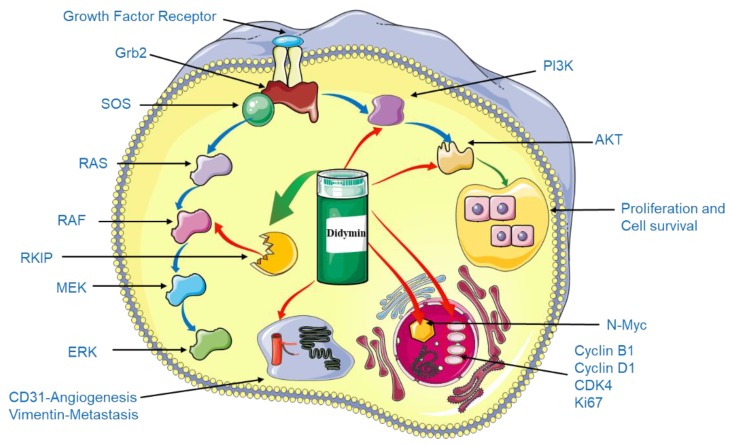
Didymin affects neuroblastoma signaling pathways. Stimulating the expression of RKIP is a key role for didymin to exert its efficacy. Also, didymin inhibits N-Myc transcription, on the other hand, didymin decreases the expression levels of PI3K, Akt, vimentin, and down-regulates cyclin D1, B1, and CDK4. By staining the pathological sections of the tumor tissue, didymin not only reduced the expression of the angiogenesis marker CD31 in vivo but also inhibited the expression of the proliferation markers Ki67 and N-Myc. The blue arrow indicates normal signal transduction, the green arrow indicates enhancement, and the red arrow represents inhibition.

Neuroblastoma is generally considered as a childhood cancer of specialized cells found in nerve tissues. The prognosis of infants with early neuroblastoma has improved significantly in the past 30 years, but the prognosis of late/older patients is still challenging. Moreover, due to the special nature of children, complementary and alternative medicine with lower side effects are more preferable in neuroblastoma treatment. Medicinal herbs are important for cancer treatment due to their multiple therapeutic targets and usually very high safety thresholds. Therefore, modern studies are increasingly looking for active anti-cancer components in safe diets, naturally derived foods such as fresh fruits, vegetables and compounds derived from them, for children’s neural and organ systems to develop normally [55]. Sing-Hai et al. studied the potential therapeutic effects of didymin in the treatment of neuroblastoma and attempted to elucidate the intracellular signaling pathways involved [56]. In this study, didymin could inhibit proliferation and induce apoptosis. Unlike other anti-cancer therapeutics for neuroblastoma, as shown in Figure 4, didymin could stimulate expression of Raf kinase inhibitory protein (RKIP) and inhibits N-Myc expression, while not involving p53 during the proapoptotic process [56]. In addition, didymin decreases the expression levels of PI3K, Akt, vimentin, and down-regulates cyclin D1, B1, and CDK4. More importantly, in vivo mice xenograft studies first investigated the in vivo anti-tumor properties of didymin and observed that didymin at a dose of 2 mg/kg bw could significantly reduce the tumor size compared with controls. Didymin holds more potential for neuroblastoma therapy with is low cost, safety, and identified efficacy. Dietary flavonoids, including didymin, have unique anticancer properties and multi-target mechanisms, which is why they have been proposed as one of the most promising therapeutic drug options for neuroblastomas in children [57,58].

Breast cancer is a malignant tumor that occurs in the glandular epithelium of the breast. It is generally believed that the breast is not an important organ to maintain the vital activity of human beings [59,60]. In situ breast cancer is not fatal, however, because breast cancer cells lose the characteristics of healthy cells, the cells are loosely connected and quickly fall off. Once cancer cells fall off, free cancer cells can spread throughout the body with blood or lymph fluid, forming a metastasis and becoming life-threatening [61]. Inspired by its anti-tumor properties, the application of didymin in breast cancer treatment has also emerged. Hsu et al. observed that didymin could effectively inhibit phthalate-mediated invasion, migration, and proliferation of breast cancer cells, especially phthalate induced tumor aggressiveness [62]. This research did not study the anti-tumor efficacy of didymin alone in breast cancer. In the alternative, the authors first studies whether phthalate, one of the main components of plastics, would promote cancer in the breast cancer tumor microenvironment. In tumor-associated mdDC (TADC)-mediated cancer, evidence showed that phthalates play a key role, with elevated proliferation, migration, and invasion [62]. TADC could create a favorable microenvironment for tumor cells by modulating several components in the cancer process. In modern society, phthalates are widely used in humans’ daily lives due to their special softening effects, and people are almost inevitably exposed to phthalates. Didymin could act as an antidote against malignant cancers caused by phthalates. Dietary therapy, including dietary flavonoids, as a safe and effective way has received widespread attention to cancer prevention. The evidence reveals that didymin as a readily available dietary flavonoid glycoside can reverse the adverse effects of environmental toxins on cancer. Though lack of more in-depth mechanism study, didymin has a therapeutic effect on tumor cells that therapeutic effects may be related to its anti-tumor, anti-inflammatory and therapeutic effects on immune cells. At present, more extensive and more in-depth treatment like dietary flavonoids with anti-tumor property is more meaningful for moderate treatment of cancer treatment/prevention. 

### 3.2. Didymin and Neuroprotective Property

Many studies have confirmed that oxidative stress may play an essential role in the pathogenesis of multiple neurodegenerative diseases, e.g., Alzheimer’s disease [63], Parkinson’s disease [64], Huntington’s disease [65], amyotrophic lateral sclerosis [66]. Oxidative stress from oxidative metabolism causes basic damages, which are mostly indirectly caused by reactive oxygen species generation, and highly associated with neurodegenerative diseases [67,68,69]. ROS, e.g., H_2_O_2_, (superoxide anion, and hydroxyl) fundamentally damage biomolecules, could cause apoptosis or cell necrosis, and ultimately lead to nerve damage. Therefore, removing excess ROS or inhibiting their production with antioxidant molecules could effectively maintain cell redox homeostasis and prevent oxidative damage. The induction of neuroprotection or neurotrophy by therapeutic agents that prevent or against progressive neurodegeneration is the most common method used today [70]. In recent years, people are increasingly looking for natural compounds with neuroprotective effects for ROS damage prevention/treatment. Fortunately, people have noticed that free radical dietary flavonoid antioxidants can be used to prevent inflammation, aging, and reduce the incidence of neurodegenerative diseases. In an elegant study, Morelli et al. showed that didymin has the effect of scavenging free radicals and the capability of rescuing the neuronal cells from oxidative damage in an in-vitro study [42]. In this study, didymin demonstrated a neuroprotective effect that prevented H_2_O_2_-induced neurotoxicity. Mechanistic studies indicated that this neuroprotective effect of didymin might be due to activation of antioxidant defense enzymes as well as to the inhibition of apoptotic features, such as p-JNK and caspase-3 activation. This result supported the notion that didymin may be a potential therapeutic agent for the treatment of neurodegenerative diseases. It is well acknowledged that neurodegenerative diseases need long-term medication. Current research indicates that didymin is a safe oral drug with potential clinical effects, and has a more significant practical application of this neurodegenerative disease, but related research is currently limited.

### 3.3. Didymin for Anxiolytic-Like and Antinociceptive Actions

Perhaps the most studied properties of flavonoids are their anti-oxidant effects, which also are indirectly associated with other health beneficial properties, such as cytoprotective activities. Reduced ROS levels could rescue injured cells from oxidative stress-induced death, implicated in abovementioned H_2_O_2_-induced neurotoxic and other several pathologies, e.g., Alzheimer’s disease. The neuropharmacological properties of flavonoids usually refer to their neuroprotective activity, while research related to anxiolytic-like and antinociceptive actions of flavonoids are rare. Estrada-Reyes et al. first studied the putative depressant effects of *Clinopodium mexicanum* extracts on the central nervous system [71]. Leaves of *Clinopodium mexicanum* have been used in the Mexican traditional medicine for sleeplessness, analgesic and sedative treatment. The aqueous extracts of leaves (AECM)-treated mice produced prolonged sleeping time, sedative effect, and delayed the onset of seizures induced by pentylenetetrazole. Chemical analysis revealed that flavonoid glycosides, including didymin, poncirin, and isonaringenin are the three main components of AECM [71]. Among them, didymin is the leading constituent of the complex mixture of flavonoids present in the active extracts of *Clinopodium mexicanum*. Therefore, in their follow-up study, Cassani et al. evaluated the in vivo sedative, anxiolytic-like and antinociceptive effects of didymin which was isolated from leaves of *Clinopodium mexicanum* [41]. Didymin showed significant anxiolytic-like activities and was able to against thermal stimuli-induced nociception, and this anxiolytic-like action could be blocked by pitrotoxin but was enhanced by muscimol. Such effects are not related to changes in locomotor activities, but instead, involved in GABAergic system participation. This study not only demonstrated that didymin possesses neuropharmacological properties, namely anxiolytic-like and antinociceptive effects, in a mice model but supports long history of traditional Mexican application of *Clinopodium mexicanum* to promote sleep and sedation.

### 3.4. Didymin for Hepatic Cytoproct Activity

Hepatic disease with critical hepatocyte damage mainly has three causes: hepatic viruses, excessive alcohol consumption, and hepatotoxins. During the hepatic injury process, acute or chronic inflammation is usually involved and identified as the hallmark of liver damage or early liver fibrosis [72,73,74]. Since didymin not only exhibits anti-inflammatory properties but also regulates the expression of PKIP, it is thought to have potential in liver injury and hepatic fibrosis treatment. Huang et al. isolated didymin from *Origanum vulgare* and investigated its role in liver injury treatment [40]. Didymin significantly reduced liver damage caused by CCl_4_ administration and was noted to decrease serum alanine aminotransferase (ALT) and aspartate aminotransferase (AST) activities. On the one hand, didymin stimulates the anti-oxidative route, down-regulates CYP2E1 activity, reduces lipid peroxidation levels, ROS and NO production and enhanced hepatic anti-oxidative enzyme activation. On the other hand, didymin reduced the expression of pro-inflammatory cytokines, such as tumor necrosis factor-α (TNF-α), interleukin-6 (IL-6) and interleukin-1β (IL-1β). Moreover, RKIP expression was notably enhanced. This group also demonstrated the didymin could alleviate hepatic fibrosis and collagen deposition in a CCl_4_-induced liver damage rat model [19]. Didymin also could significantly reduce mitochondrial membrane potential. The EPR/MARK and P13k/Akt pathways were both inhibited by didymin via RKIP expression regulation. Collectively, the above evidence suggests that didymin may be a novel hepatoprotective agent for the future clinical treatment of liver fibrosis and liver injuries.

### 3.5. Didymin and Cardiovascular Activities

Diabetic patients are usually affected by a higher risk of heart disease and myocardial infarction [75]. The hyperglycemia condition increased the inflammation, and oxidative stress also deteriorates endothelial cell dysfunction [69,76]. Therefore, therapeutic agents with anti-inflammatory and anti-oxidative stress effects have clinical potential in endothelial dysfunction and related cardiovascular complications. With a long history, flavonoids have drawn attention to cardiovascular complications with their multiple therapeutic targets [77]. Shukla et al. studied the preventive effects of didymin in cardiovascular diseases, especially the preventive effect on endothelial dysfunction [78]. Didymin pretreatment could prevent the high glucose (HG)-induced lower cell viability of human umbilical vein endothelial cells (HUVECs). Several mechanisms contribute to the rescue benefits of didymin, including reduced generation of ROS and lipid peroxidation products, limited HG-induced eNOS decrease, and iNOS expression increase, and reducing the adhesion of monocytes to endothelial cells. Didymin also showed strong anti-inflammatory properties, inhibiting the expression of different inflammatory cytokines and chemokines in HG-treated HUVECs. It is worth mentioning that compared to other flavonoids like rutin and the commonly used natural active ingredient curcumin, the dose of didymin required in endothelial dysfunction prevention is quite low. The joint effect of its anti-inflammatory and antioxidant properties can explain the endothelial recovery properties of the flavanone didymin. Endothelial dysfunction is one of the major pathological processes of atherogenesis. Therefore, didymin may shortly become a potential natural therapeutic agent for the treatment of cardiovascular complications caused by hyperglycemia.

In this section, we have summarized several potential therapeutic applications of didymin in various diseases. It is noticeable that as a dietary flavonoid, didymin shows various health beneficial properties, including anti-oxidant, anti-tumor, anti-inflammatory, cytoprotective and cardiovascular-protective properties. The molecular mechanisms behind these therapeutic effects vary considerably (Table 2). 

## 4. Conclusions and Future Perspectives

Currently, there is increasing interest in the potential benefits of complementary and alternative medicines [79]. There is mounting evidence that flavonoids are rich in bioactive compounds with therapeutic properties. The scientific knowledge about the dietary flavonoid didymin has been corroborated by emerging investigations conducted in the last decade, especially the last five years. Of the most noticeable therapeutic influences of didymin, researchers have mostly pointed to the anti-oxidant potential and anti-tumoral activities through different regulatory effects on the molecular targets involved. Didymin, due to its therapeutic effects and an excellent safety profile, was demonstrated to be a potential candidate for the prevention and treatment of some diseases. Researchers have found out that in polyphenol redox systems, the solvent plays a critical role inthe bioactivities of natural extracted antioxidants [80]. Moreover, their therapeutic activities might differ in different surroundings, for example, in hydrophilic environments, flavonoids such as neohesperidin, hesperidin, hesperetin, didymin, and isosakuranetin all possess antioxidant properties, while in a lipophilic environment, such antioxidant activity is generally decreased. Although didymin may be a promising flavonoid that is therapeutically active in the prevention and treatment of various diseases, in vivo data are still scarce and most in vitro data was obtained in physiologically-irrelevant conditions. In addition, the mechanisms behind the action of didymin are not comprehensively understood. 

On the other hand, low bioavailability might be an obstacle to the use of flavonoids as drugs or health products. Usually, free flavonoids are quickly dissolved in methanol, ethanol and other organic solvents, but insoluble or almost insoluble in water. Flavanones are significant dietary components and are also considered to have poor bioavailability, as very few phase II metabolites are detected in the bloodstream. When various metabolites and catabolites which were previously ignored are taken into consideration, flavanones and anthocyanins might be more absorbable than previously believed [81], but still, to meet the desired therapeutic dose, proper formulation strategies are need to enhance the solubility and oral absorption. One successful strategy for increasing the bioavailability of flavonoids is to increase their hydrophilicity through the use of microemulsions, lecithin complexation, and polyvinylpyrrolidone dispersions. In recent years, glycosylation of flavonoids has also become a conventional method. Glycosylation is an also important pharmaceutical method for optimizing the pharmacokinetic and pharmacodynamic properties of small molecule drugs, mainly by regulating the solubility, stability, bioavailability and biological activity of the compounds [82]. Nanoformulation, e.g. using nanocrystals [83,84] and nanoparticles [85], might also provide new prevention and treatment options for naturally extracted flavonoids, like didymin. Cyclodextrin is an excipient that is non-toxic and has no side effects. It also can increase the solubility and bioavailability of a drug after forming an inclusion compound [86]. Moreover, it has been reported that liposomes can be used as a carrier for the administration of flavonoids, which could increase the solubility of drugs, prolong the action time, and have a sustained release effect [87].

Didymin is still in the initial stage of laboratory research and development, and there is still no consistent clinical data, but shortly, didymin may provide more effective approaches in the treatment of complex diseases through different formulation methods.

## Figures and Tables

**Table 1:** The source and extraction separation method of didymin.

Didymin is a Flavonoid Glycoside Commonly Found in Citrus Fruits	
Source	Orange [20]
	Grapefruit [21,38]
	Mandarin [39]
	Bergamot [19]
	Other citrus [17,26,27]
	*Origanum Vulgare* [40]
	*Clinopodium* [22,41] etc.
**HPLC is the Preferred Method for Separating and Detecting Citrus Flavonoids**	
Extraction and Detection Method	MS-HPLC [28,29]
	Ultra-HPLC (UHPLC) [30,31,32]
	Comprehensive multidimensional LC methods [33]
	RP-HPLC and photodiode array detection [23]
	Nano-LC/UV-Vis apparatus [34]
	UAE [35]
	UPLC-ESI-QTOF-MS/MS [36]

**Table 2:** Different diseases and mechanism study related to didymin.

Disease	Mechanism Studies	Ref.
Lung cancer	The primary pathway of apoptosis induced by didymin is the Fas/Fas ligand apoptotic system, which does not mediate p53 and p21/WAF1.	[17]
Neuroblastoma	Inhibition of N-Myc transcription, up-regulated RKIP and down-regulated PI13K, Akt and vimentin.	[56]
	Downregulation of cyclin D1, cyclin B1, CDK4, CD31, Ki67, and N-Myc also enhance the anti-tumor effect of didymin.	
Breast cancer	Didymin can effectively inhibit phthalate-mediated invasion, migration, and proliferation of breast cancer cells.	[62]
Neurodegenerative disease	Removing excess ROS or inhibiting its production by antioxidant molecules could effectively maintain cell redox homeostasis and prevent oxidative damage.	[42]
	Effectively inhibits apoptosis and activates antioxidant defense enzymes.	
Sleeplessness	GABAergic system participation in the anxiolytic actions of didymin. Didymin could exert its anxiolytic-like effect through the interaction with the GABAA receptors.	[41]
Hepatic diseases	Didymin has antioxidant activity, scavenges free radicals, and regulates MAPK and NF-κB signaling pathways.	[19]
Cardiovascular complications	Didymin prevented HG-induced (ROS) and the production of lipid peroxidation product malondialdehyde and prevented HG-induced monocyte-endothelial cell adhesion, ICAM-1 and VCAM-1 expression, and NF-κB activation.	[78]
	Didymin inhibits the release of various inflammatory cytokines and chemokines from HG-treated HUVECs.	
